# Revolutionizing orthopedic healthcare: a systematic review unveiling recombinant antimicrobial peptides

**DOI:** 10.3389/fmicb.2024.1370826

**Published:** 2024-04-29

**Authors:** Vincenzo Pennone, Elena Rosini, Elena Mascheroni, Silvia Gianola, Greta Castellini, Silvia Bargeri, Arianna B. Lovati

**Affiliations:** ^1^Cell and Tissue Engineering Laboratory, IRCCS Istituto Ortopedico Galeazzi, Milan, Italy; ^2^Department of Biotechnology and Life Sciences, University of Insubria, Varese, Italy; ^3^Unit of Clinical Epidemiology, IRCCS Istituto Ortopedico Galeazzi, Milan, Italy

**Keywords:** orthopedic infections, antibiotic resistance, recombinant antimicrobial peptides, yield, minimum inhibitory concentration

## Abstract

The increasing demand for orthopedic surgeries, including joint replacements, is driven by an aging population and improved diagnosis of joint conditions. Orthopedic surgeries carry a risk of infection, especially in patients with comorbidities. The rise of antibiotic resistance exacerbates this issue, necessitating alternatives like *in vitro* bioengineered antimicrobial peptides (AMPs), offering broad-spectrum activity and multiple action mechanisms. This review aimed to assess the prevalence of antimicrobial potential and the yield after purification among recombinant AMP families. The antimicrobial potential was evaluated using the Minimum Inhibitory Concentration (MIC) values against the most common bacteria involved in clinical infections. This systematic review adhered to PRISMA guidelines, focusing on *in vitro* studies of recombinant AMPs. The search strategy was run on PubMed, Scopus and Embase up to 30^th^ March 2023. The Population, Exposure and Outcome model was used to extract the data from studies and ToxRTool for the risk of bias analysis. This review included studies providing peptide production yield data and MIC values against pathogenic bacteria. Non-English texts, reviews, conference abstracts, books, studies focusing solely on chemical synthesis, those reporting incomplete data sets, using non-standard MIC assessment methods, or presenting MIC values as ranges rather than precise concentrations, were excluded. From 370 publications, 34 studies on AMPs were analyzed. These covered 46 AMPs across 18 families, with Defensins and Hepcidins being most common. Yields varied from 0.5 to 2,700 mg/L. AMPs were tested against 23 bacterial genera, with MIC values ranging from 0.125 to >1,152 μg/mL. Arenicins showed the highest antimicrobial activity, particularly against common orthopedic infection pathogens. However, AMP production yields varied and some AMPs demonstrated limited effectiveness against certain bacterial strains. This systematic review emphasizes the critical role of bioengineered AMPs to cope infections and antibiotic resistance. It meticulously evaluates recombinant AMPs, focusing on their antimicrobial efficacy and production yields. The review highlights that, despite the variability in AMP yields and effectiveness, Arenicins and Defensins are promising candidates for future research and clinical applications in treating antibiotic-resistant orthopedic infections. This study contributes significantly to the understanding of AMPs in healthcare, underscoring their potential in addressing the growing challenge of antibiotic resistance.

**Systematic review registration:**https://osf.io/2uq4c/.

## 1 Introduction

The demand for arthroplasty and orthopedic surgeries involving implants has shown a consistent upward trend in recent years, driven by various factors. These factors encompass an aging population and heightened awareness and diagnosis of joint-related ailments. According to data sourced from the American Joint Replacement Registry in 2019, roughly 1.8 million total joint replacement procedures were performed. Among these, total knee replacements accounted for approximately 1 million procedures, while total hip replacements constituted around 600.000 procedures (Levine et al., 2020). This trajectory is expected to persist, given the aging demographic and the increasing need for joint replacement procedures (Shichman et al., 2023).

Beyond joint replacements, there exists a spectrum of orthopedic surgeries necessitating implants such as fracture fixation, spinal fusion, and arthroplasty of shoulder or elbow. Collectively, these surgeries carry the potential risk of orthopedic infections, particularly in patients with underlying comorbidities such as diabetes, cardiovascular issues, or compromised immune systems (Lai et al., 2007).

Orthopedic infections can lead to substantial morbidity and complications when not effectively treated. Furthermore, the rise of antibiotic resistance is becoming an increasingly pressing issue within the realm of orthopedic infections and healthcare at large, akin to a silent pandemic (Salam et al., 2023). The excessive and inappropriate utilization of antibiotics has played a pivotal role in fostering the emergence of antibiotic-resistant bacterial strains, presenting a formidable clinical challenge. As such, there is an immediate and imperative need for intervention to address this critical concern.

Antimicrobial peptides (AMPs) are naturally occurring molecules that play a crucial role in the innate immune system. AMPs have gained significant attention as potential alternatives to traditional antibiotics in the fight against antibiotic-resistant infections, since exhibit: (i) broad-spectrum antimicrobial activity against bacteria (Gram-positive and Gram-negative), viruses (enveloped and non-enveloped), yeasts, fungi, molds, and parasites (Zasloff, 2002; Radek and Gallo, 2007; Marxer et al., 2016); (ii) multiple mechanisms of action on different biological targets to traditional antibiotics and distinct pathways, thus decreasing the propensity for resistance to occur (Hancock and Chapple, 1999; Zasloff, 2002); (iii) regulation of key immunomodulatory mechanisms in the innate immune system (Al-Rayahi and Sanyi, 2015; Zhang and Gallo, 2016).

AMPs show significant chemical diversity in nature, at the same time exhibiting common structural properties. These peptides usually are less than 100 amino acids long, especially consisting of positively charged (i.e., lysine, arginine, and histidine) and hydrophobic residues, these latter accounting for more than 50% (Jenssen et al., 2006). AMPs are commonly classified based on their secondary structure into four different groups: α-helical, β-sheet, mixed, and cyclic structures. Their structural organization is crucial for the interaction with biological targets. The amphiphilic α-helix promotes the interaction with cell membranes, thus allowing membrane disruption; in particular, the interaction is facilitated by the presence of cationic and hydrophobic residues on the opposite faces of the helix motif (Wiradharma et al., 2013). The β-sheet conformation shows amphipathic properties due to the presence of the spatially defined polar and non-polar domains in at least a pair of two β-strands linked by disulphide bridges conferring stabilization to the overall peptide structure (Lee et al., 2016; Kumar et al., 2018). The supplemental head-to-tail cyclization further increases the stability of the secondary structure, without the need of additional conformational changes upon the interaction with cell membranes. Three or four disulphide bridges stabilize the α-helix/β-sheet mixed structures, that consist of positively charged residues arranged in the helix and of hydrophobic amino acids in the β-sheet motif (Yang, 2012).

The amphipathic nature of the overall AMPs is essential for their ability to interact with bacterial membranes. The antimicrobial activity is mainly exerted through two different mechanisms: (i) interaction with bacterial cell membrane to impair its structural integrity; (ii) interaction with intracellular targets to inhibit the synthesis of nucleic acids, key enzymes and functional proteins. The physicochemical properties of AMPs promote the initial interaction with the cell surface resulting in the disruption of membrane integrity: in detail, the first essential step of interaction is represented by the electrostatic binding of the positively charged residues of AMPs to the anionic lipids of bacterial cell membrane (Bahar and Ren, 2013; Kumar et al., 2018). The subsequent insertion into the hydrophobic core of the bilayer is mediated by the interaction of hydrophobic amino acids of AMPs with the fatty acyl chains of membrane lipids (Pirtskhalava et al., 2021). Once the AMPs critical aggregation concentration on the membrane is reached, the membrane disruption occurs mainly through three mechanisms. In the “barrel-stave model,” the AMPs laterally accumulated on the membrane, rotate perpendicularly to the plasma membrane, thus forming a channel (López-Meza et al., 2011). AMPs are inserted perpendicularly in the hydrophobic region of the bilayer through a peptide-lipid complex in the “toroidal model,” thus promoting a local membrane curvature and forming a toroidal pore (Hazam et al., 2019). In the “carpet model,” at first AMPs are bound parallel to the cell surface thanks to the electrostatic interactions; once the critical concentration is reached, AMPs form micelles resulting in membrane disruption (Hazam et al., 2019).

Many AMPs exert their antimicrobial activity through a non-membrane targeting mechanism: the inhibition of protein synthesis by interacting with the ribosome, the interaction with nucleic acids, the binding with the precursor lipid II crucial for the peptidoglycan synthesis, the interaction with different chaperone proteins to block the protein folding pathway, resulting in bacterial death (Otvos et al., 2000; Brogden, 2005; Essig et al., 2014; Mardirossian et al., 2018).

Lastly, AMPs have the potential to be used in combination with existing antibiotics, enhancing their efficacy and reducing the likelihood of resistance development.

In the context of orthopedic infections, AMPs show promise as potential therapeutic agents. They can be used as topical agents to prevent and/or treat surgical site infections, or they can be incorporated into biomaterials used with orthopedic implants to reduce the risk of infection (Li et al., 2023). While AMPs hold great potential, there are still challenges to overcome before they can be widely used in clinical practice: higher stability, reduced toxicity, and optimized production methods. The scale up of AMPs production is crucial for their application to serve market requirements. Three strategies can be applied to obtain AMPs for clinical use: direct extraction from natural sources or bioengineering them through chemical synthesis and recombinant production. Up to now, few AMPs have been isolated from natural producers such as plants, insects, bacteria by applying a number of laborious and expensive extraction and purification steps (Moreira et al., 2011; Tang et al., 2018). Chemical synthesis and recombinant production of AMPs represent two conventional optional methods.

Chemical synthesis of AMPs involves stepwise assembly of amino acids, enabling precise control over sequence design and offering customization. Recombinant AMP production, utilizing genetic engineering in microorganisms, offers scalability in large-scale manufacturing but may require additional purification steps. To be optimal, the production method should be industrially scalable, reproducible, biocompatible, following the good manufacturing practices and at low cost.

In chemical production, solid-phase-peptide synthesis is the most common method used, also at industrial scale (Merrifield, 1963). The N-terminal amino acid is bound to a polystyrene resin via the carboxyl end. Coupling compounds are used to bind amino acids to each other, chemically modified with protecting groups at their N-terminus or side chains. The process is repeated to obtain the desired final peptide. The two most used N-protecting groups are fluorenylmethoxycarbonyl (Fmoc) and tert-butoxycarbonyl (Boc) (Pedersen et al., 2012). These latter substances, coupled with the use of dimethylformamide and dichloromethane in the chemical method, are environmentally hazardous, thus rendering the overall process ecologically unsustainable (Lawrenson et al., 2017). The automation and improvement in instrumentation allowed to produce peptides up to 50 amino acids. The simplicity and the fast optimized protocol allowed to mass produce AMPs. However, the incorporation of residues such as cysteine, aspartic acid, and histidine, as well as the production of glycopeptides still remain a great challenge (Pedersen et al., 2012). Moreover, an important limitation is represented by the huge need for starting materials (Kaur, 2018). Noteworthy, the D-enantiomer of amino acids can be incorporated during the chemical synthesis, resulting in peptides not recognized by proteases and the immune system, thus allowing to increase their *in vivo* stability (Bland et al., 2001). At the same time, the incorporation of D-enantiomers could modify the secondary structure of AMPs, potentially resulting in a lower antibacterial activity (Lee et al., 2004). Despite the possibility to scale up the chemical production, the overall process is still costly when compared with the recombinant production in a bacterial host. In this latter case, to reduce toxicity and proteolytic degradation processes, recombinant AMPs are mainly produced as fusion proteins (Vassilevski et al., 2008; Li, 2009; Costa et al., 2014).

The choice of the host, expression plasmid, and fusion tags strongly influence the production yield. In this context, bacteria, yeasts, and plants represent the most common expression systems used. In particular, *Escherichia coli* is the most preferred recombinant system, mainly due to its rapid growth and well-known genetic and biochemical properties (Ingham and Moore, 2007): high expression levels and low fermentation cost resulted in high AMPs production yields (Meng et al., 2016; Ashcheulova et al., 2018). In addition, *Bacillus subtilis* has been employed as host, mainly for the possibility to reach high cell density, the absence of endotoxins and the secretion of the produced AMPs in the culture medium, thus simplifying the downstream purification process. Noteworthy, several strategies can be applied from a molecular point of view, as well as acting on protein expression conditions, to optimize AMPs production. In addition, protein engineering approaches such as site-directed and site-saturation mutagenesis, can be employed to modify specifically the amino acid sequence or to generate small peptide libraries to be screened for the required antimicrobial activity.

Based on all these assumptions, the authors included in the current systematic review only the AMPs recombinantly expressed, considering the low amounts of peptides obtained by extraction from natural sources and the high costs, technical limitations and environmental issues that may arise from the chemical synthesis. Based on advancements in protein expression platforms and fusion proteins approaches, the recombinant production represents now a solid option to make AMPs accessible at low cost and high yield for clinical applications.

This review aims to assess the prevalence of antimicrobial potential and the production yield (after purification) among recombinant AMP families. The antimicrobial potential was evaluated using the Minimum Inhibitory Concentration (MIC) values against the most common bacteria involved in clinical infections.

## 2 Materials and methods

### 2.1 Study design

This systematic review follows Preferred Reporting Items for Systematic Reviews and Meta Analysis (PRISMA) framework guidelines. The study protocol is registered in OSF repository, available at the following link: https://osf.io/2uq4c/.

### 2.2 Question

This review aims to assess the prevalence of antimicrobial potential and the production yield (after purification) among recombinant AMP families. The antimicrobial potential was evaluated using the MIC values against the most common bacteria involved in clinical infections.

### 2.3 Eligibility criteria

A Population, Exposure and Outcome (PEO) model was developed to extract information from the studies. Table 1 summarizes the data extracted from the studies included in the analysis. The inclusion criteria were:

- *In vitro* studies;
- Production of recombinant AMPs;
- Availability of the yield of the purified peptide;
- MIC values of the AMPs against pathogenic bacteria.

**Table 1:** PEO model.

Population	Bacterial pathogens
Exposure	Recombinant AMPs
Outcome	Antimicrobial activity (MIC) and production yield

Exclusion criteria for the PEO model were:

- Full text not available;
- Studies not in English language;
- Reviews and conference abstracts;
- Books and book chapters;
- Studies of chemical synthesis of the AMPs that report only one of the two data sets (yield and MIC);
- The utilization of AMPs that are not purified;
- MIC values assessed using a protocol different from the ISO 20776-1 standard (broth microdilution method) or the Disk diffusion method according to CLSI or EUCAST guidelines (EUCAST, 2024);
- MIC values reported as ranges, not as precise concentration.

### 2.4 Search strategy

A search strategy was run on PubMed, Scopus and Embase up to 30^th^ March 2023. The keywords searched and combined with AND were “antimicrobial peptide” and “recombinant expression.” No restriction was applied. On Scopus, the string used was TITLE-ABS-KEY (“antimicrobial peptide” AND “recombinant expression”). Additionally, the EMBASE database was also screened with keywords: (“antimicrobial peptide”/exp. OR “antimicrobial peptide”) AND “recombinant expression”.

### 2.5 Data collection and analysis

#### 2.5.1 Selection of studies

Two investigators (VP and ABL) independently performed a literature search and screened the references based on title and abstract. The eligible papers were selected based on the full text and only the studies matching the inclusion criteria were finally included. Any disagreement was discussed and solved. All the references obtained from the three databases were placed in a single Excel file and ordered by Title. The first step of selection was to manually remove duplicate entries. Subsequently, the review papers, conference abstracts, papers in a language different from English, and papers with full text not available were removed. For a more in-depth analysis of the PEO model, the remaining full-text manuscripts were screened.

#### 2.5.2 Data collection and management

Two investigators (VP and ABL) independently performed the data extraction phase. Any disagreement was solved by consensus. The following general characteristics of the included studies were extracted: authors, title and year of publication. Then, outcome data about the antimicrobial activity (MIC value) and the production yield were collected using a pre-defined data collection form in an Excel sheet. We piloted the data extraction form on a sample of ten studies to capture all important features. Thus, using the final collection form containing raw data, the AMP yields were recorded in mg/L. If the unit of measurement was different, a conversion was made to report the yield as mg/L. The MIC values (μg/mL) were intended to be the minimal concentration that inhibited the growth of the tested microorganism. If the MIC values were reported as μM, the following conversion was used: μg/mL = μM*kDa. Other information was added to the table including the raw data: expression system, AMP family, AMP structure, microorganism genus and MIC category. To easily distinguish between yeast and prokaryotic plasmids, the expression system host was introduced. The AMP family was either found in the paper or searched online (Protein Data Bank PDB, Antimicrobial Peptide Database APD, PubMed, last accessed on October 5^th^ 2023). The AMP structure was either obtained by PDB or estimated with AlphaFold2 Colaboratory (last accessed on October 5^th^ 2023). The microorganism genus was added to categorize all the different species and strains of the same genus. Since the MIC value was not always reported as continuous numbers, but also as “<” or “>” values, a statistical comparison was not possible. However we introduced the MIC category, intended to categorize the MIC values of each AMP family by arbitrarily dividing MIC values into two groups based on the 75^th^ percentiles of each AMP family. Specifically, one group had MIC values that were less than or equal to the 75^th^ percentile, and the other group had MIC values that were greater than the 75^th^ percentile.

#### 2.5.3 Assessment of risk of bias (ROB) in included studies

The *in vitro* section of the Toxicological data Reliability assessment Tool (ToxRTool, Schneider et al., 2009) was used for the ROB assessment. The tool included 18 criteria, divided into 5 groups: “Test substance identification,” “Test system characterization,” “Study design description,” “Study results documentation,” and “Plausibility of study design and results.” All criteria were answered assigning a score of “0” for unmet criteria or “1” for met criteria. By answering all 18 criteria, the tool calculated the total score and assigned a data reliability of “reliable without restriction” (15–18 points), “reliable with restrictions” (11–14 points) or “not reliable” (<11 points). Additionally, the tool considered 6 of the 18 criteria, called “red criteria”, as minimum elements for a study to be considered reliable. Only if all red criteria were met, i.e., rated as ‘1’, the tool assigned a data reliability of “reliable without restriction” or “reliable with restrictions.” If one or more red criteria were not met, i.e., rated as ‘0’, the tool assigned a data reliability of “not reliable.” The tool provided freedom to the evaluator to deviate from the categorization, if a justification was provided.

#### 2.5.4 Synthesis methods

General characteristics of the included studies were descriptively tabulated based on the PEO model. The outcome was described as association between the AMP families and the target bacterial genera represented in an alluvial chart using the online tool www.rawgraphs.io (Mauri et al., 2017). Subsequently, the most represented AMP families underwent further investigation using the ggplot2 package in R-Studio (version: 2023.06.1 + 524), and their yields and MICs against the targeted bacterial genera were visualized with alluvial charts. Eventually, between the targeted bacteria, the bacterial genera more involved in orthopedic infections (*E. coli, Pseudomonas*, and *Staphylococcus*) were analyzed to assess the AMP family more effective against them, based on the literature considered for this study.

## 3 Results

### 3.1 Summary of results

**Figure 1:**
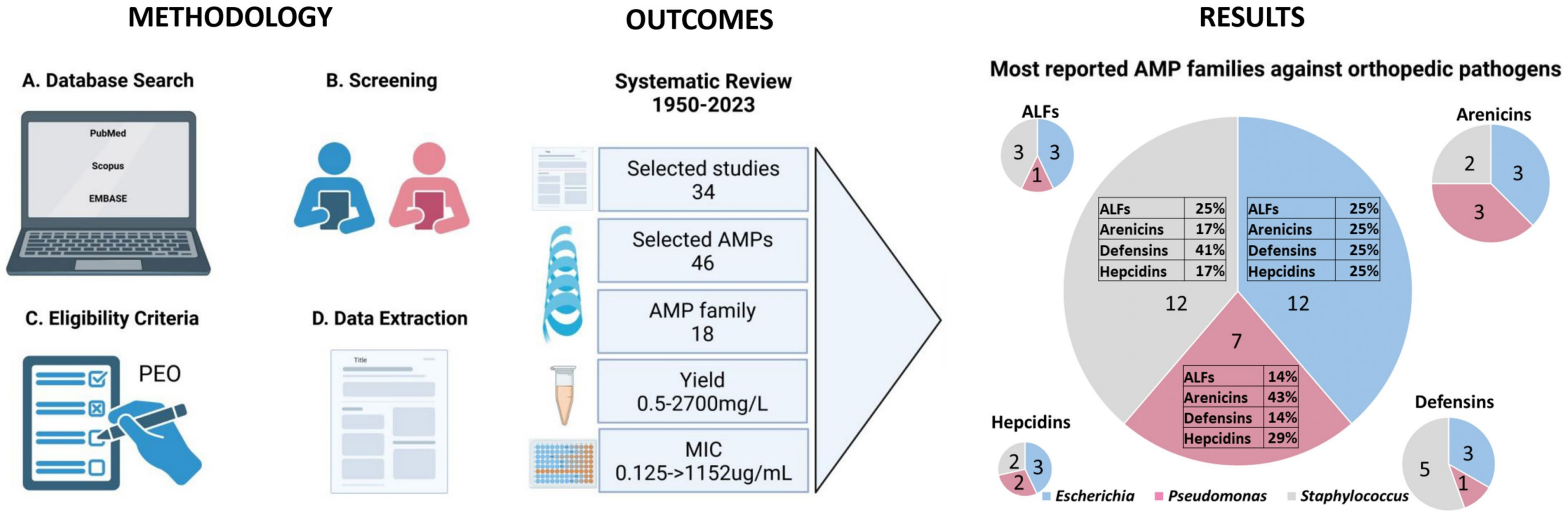
Schematic flow chart of the systematic review. The figure presents a detailed flow chart outlining the methodology from the data search to extraction. Outcomes include the selected studies highlighting the range values of yield and MIC. The figure also depicts the results, quantified by the number of selected studies, of the predominant AMP families against the three primary bacteria genera responsible for orthopedic infections. Created with BioRender.com.

**Figure 2:**
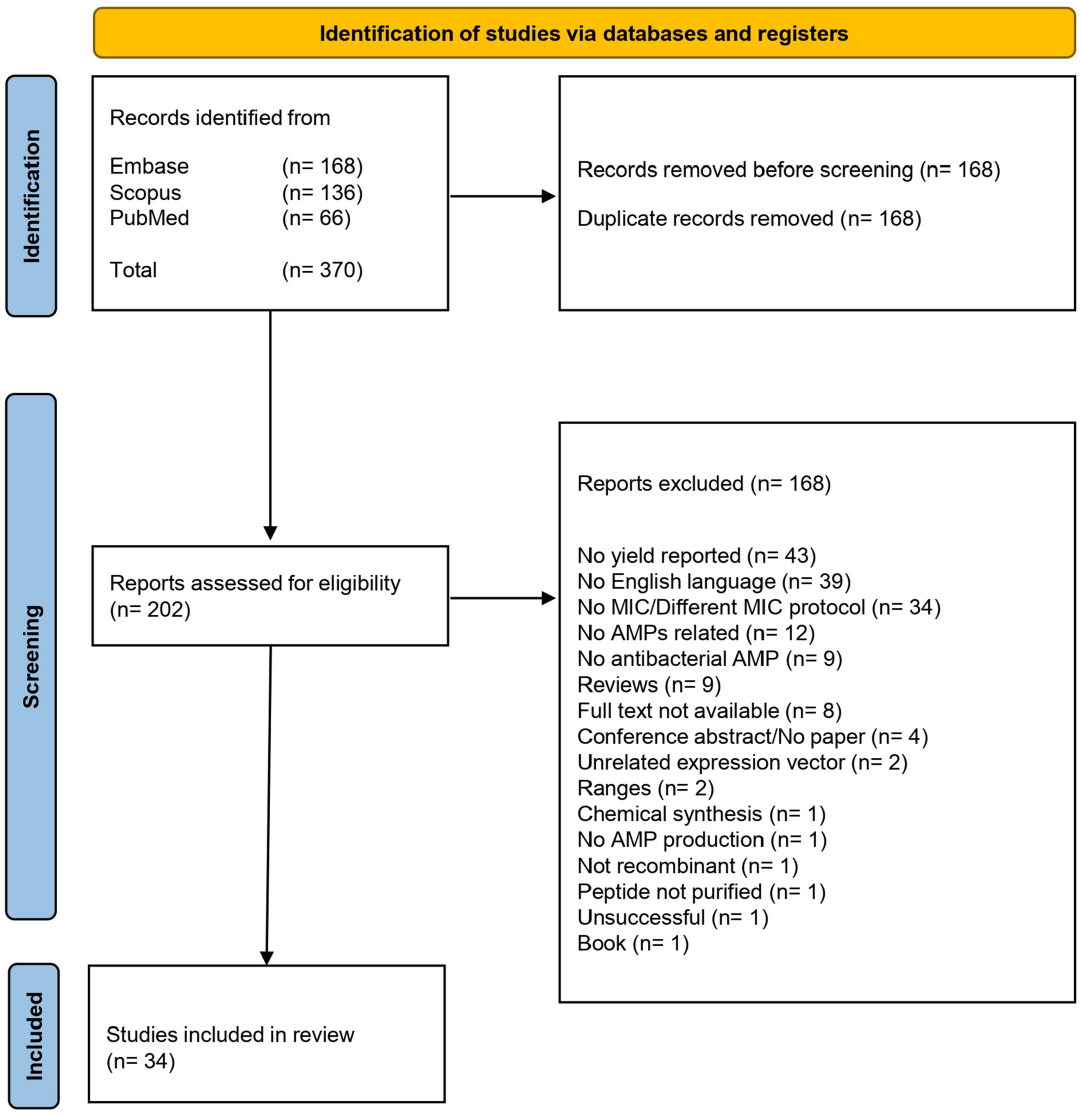
This figure illustrates the meticulous process undertaken to select studies for inclusion in our review, emphasizing our commitment to transparency and rigor. The PRISMA flow began with a comprehensive search and meticulous screening of a vast literature. After removing non-compliant studies, the data from 34 publications were extracted for the further steps of this review.

A schematic flow chart of the systematic review is represented in Figure 1. A total of 370 publications was recovered from the three different databases, as shown in the PRISMA flow chart in Figure 2. After removing 168 duplicates, 202 publications were included for the screening phase. Full text articles were analyzed, and 168 more articles were excluded since not compliant with the PEO model. Ultimately, 34 papers were analyzed in this study.

### 3.2 Characteristics of included studies

The characteristics of the AMPs described in the 34 papers considered in this analysis are summarized in Table 2, while the complete dataset is stored on OSF at the following link: https://osf.io/2uq4c/.

**Table 2:** Characteristics of the AMPs considered in this study, grouped per AMP families, in alphabetical order.

AMP Family	AMPs	AMP Structure	Microorganism	Ratio of hydrophilic residues / total number of residues (%)	Net charge at pH = 7.0	Number of Residues	Reference
Anti-lipopolysaccharide factors (ALFs)	rMnALF4 rFcALF5 mFcALF2	Mixed^1^	*Aeromonas, Agrobacterium, Bacillus, Burkholderia, Enterobacter, Escherichia, Kluyvera, Microbacterium, Micrococcus, Proteus, Pseudomonas, Salmonella, Serratia, Sporosarcina, Staphylococcus, Vibrio*	rMnALF4: 37 rFcALF5: 37 mFcALF2: 37	rMnALF4:4.10 rFcALF5:1.90 mFcALF2:8.01	rMnALF4: 99 rFcALF5: 99 mFcALF2: 98	Yang et al. (2015), Yang H. et al. (2016), and Tang et al. (2020)
Apidaecins	Apidaecins (Aps) 2	Random coil^1^	*Escherichia, Pseudomonas, Salmonella*	33	5,09	18	Mo et al. (2018)
Arenicins	rN2 15N-labeled Ar-1[V8R] 15N-labeled Ar-1 NZ17074	rN2: β-sheet. PDB code: 5Y0J^2^ Arenicin-1: β-sheet. PDB code: 2JSB^2^ Arenicin-1 V8R: β-sheet. PDB code: 5M9U^2^ NZ17074: β-sheet^1^	*Bacillus, Escherichia, Klebsiella, Listeria, Pseudomonas, Salmonella, Staphylococcus, Streptococcus*	rN2: 33 Arenicin-1:29 Arenicin-1 V8R: 33 NZ17074: 32	rN2: 3.91 Arenicin-1:5.91 Arenicin-1 V8R: 6.91 NZ17074: 3.91	rN2: 21 Arenicin-1: 21 Arenicin-1 V8R: 21 NZ17074: 22	Wang et al. (2014), Yang N. et al. (2016), Panteleev et al. (2017)
ASABF-like	HKABF	Mixed^1^	*Morganella, Staphylococcus, Vibrio*	31	6.64	49	Wang et al. (2008)
Bacteriocins	Beta Casein-E 50–52 (BCN-E 50–52) Lacticin Q	Beta Casein-E 50–52 (BCN-E 50–52): Mixed^1^ Lacticin Q: α-helix. PDB code: 7P5R^2^	*Enterococcus, Escherichia, Listeria, Salmonella, Staphylococcus*	BCN-E 50–52: 28 Lacticin Q: 34	BCN-E 50–52: 1.73 Lacticin Q: 6.00	BCN-E 50–52: 39 Lacticin Q: 53	Yu et al. (2013) and Fahimirad et al. (2017)
Cathelicidins	Fowlicidin-2 Indolicidin	Fowlicidin-2: α-helix. PDB code: 2GDL^2^ Indolicidin: Random coil. PDB code: 1G89^2^	*Bacillus, Escherichia, Listeria, Pseudomonas, Salmonella, Staphylococcus*	Fowlicidin-2: 42 Indolicidin: 23	Fowlicidin-2: 10.00 Indolicidin: 3.00	Fowlicidin-2: 31 Indolicidin: 13	Morin et al. (2006), Feng et al. (2015), and Xing et al. (2016)
Crustins	rCshFc rCruFc	AlphaFold2 Colaboratory was not able to predict a structure	*Aeromonas, Bacillus, Escherichia, Klebsiella, Micrococcus, Staphylococcus, Vibrio*	rCshFc: 22 rCruFc: 25	rCshFc: 1.27 rCruFc: 0.56	rCshFc: 87 rCruFc: 117	Zhang et al. (2007)
Defensins	Plectasin CeHS-1 CeHS-1 GP Cryptdin-2 MutantE18C-Cryptdin-2 Aurelin BmTXKS2 rMdde rCgDef	Plectasin: Mixed. PDB code: 3E7U^2^ CeHS-1: α-helix^1^ CeHS-1 GP: α-helix^1^ Cryptdin-2: β-sheet^1^ MutantE18C-Cryptdin-2: β-sheet^1^ Aurelin: α-helix. PDB code: 2LG4^2^ BmTXKS2: Mixed^1^ rMdde: Mixed^1^ rCgDef: mixed. PDB code: 2B68^2^	*Bacillus, Enterococcus, Escherichia, Klebsiella, Listeria, Micrococcus, Staphylococcus*	Plectasin: 34 CeHS-1: 45 CeHS-1 GP:38 Cryptdin-2: 36 MutantE18C- Cryptdin-2: 33 Aurelin: 43 BmTXKS2: 49 rMdde: 25 rCgDef: 35	Plectasin: 1.91 CeHS-1: 2.09 CeHS-1 GP: 5.09 Cryptdin-2:7.82 MutantE18C-Cryptdin-2:8.77 Aurelin: 4.91 BmTXKS2:2.08 rMdde: 2.91 rCgDef: 3.73	Plectasin: 41 CeHS-1: 38 CeHS-1 GP:37 Cryptdin-2: 36 MutantE18C-Cryptdin-2: 36 Aurelin: 40 BmTXKS2: 39 rMdde: 40 rCgDef: 43	Wang et al. (2006), Shenkarev et al. (2012), Chen et al. (2015), Kaur et al. (2020), Erdem Büyükkiraz and Kesmen (2022), Erviana et al. (2022), and Taghizadeh et al. (2022)
GASA/GAST	SN2	α-helix^1^	*Agrobacterium, Escherichia, Micrococcus, Staphylococcus*	33	7.64	66	Herbel et al. (2015)
Hepcidins	Sal1 Sal2a Sal2b Hepc25 ECproHep3 6x His-Factor Xa-NG-hepcidin 6x His-TEV-hepcidin	Sal1: β-sheet^1^ Sal2: β-sheet^1^ Hepc25: β-sheet. PDB code: 1M4F^2^ ECproHep3: Mixed^1^ 6x His-Factor Xa-NG-hepcidin – 6x His-TEV-hepcidin: β-sheet^1^	*Aeromonas, Bacillus, Corynebacterium, Escherichia, Micrococcus, Pseudomonas, Shigella, Staphylococcus, Vibrio*	Sal1: 20 Sal2: 32 Hepc25: 20 ECproHep3:37 hepcidin: 27	Sal1: 0.82 Sal2: 2.82 Hepc25: 1.82 ECproHep3: 3.08 hepcidin: 3.82	Sal1: 25 Sal2: 25 Hepc25: 25 ECproHep3:65 hepcidin: 26	Greenshields et al. (2008), Srinivasulu et al. (2008), Qu et al. (2013), and Janakiraman et al. (2015)
Hybrid peptides	LF15-CA8 Hybrid Magainin–Thanatin rCgPrp-CgDef Sericincecropin B	LF15-CA8: α-helix^1^ Hybrid Magainin–Thanatin: β-sheet^1^ rCgPrp-CgDef: Mixed^1^ Sericincecropin B: AlphaFold2 Colaboratory was not able to predict a structure.	*Bacillus, Escherichia, Listeria, Pseudomonas, Salmonella, Staphylococcus*	LF15-CA8: 47 Hybrid Magainin–Thanatin: 34 rCgPrp-CgDef: 41 Sericincecropin B: 49	LF15-CA8:7.95 Hybrid Magainin–Thanatin: 7.00 rCgPrp-CgDef: 5.74 Sericincecropin B: 0.01	LF15-CA8: 30 Hybrid Magainin–Thanatin: 29 rCgPrp-CgDef: 81 Sericincecropin B: 136	Feng et al. (2014), Tian et al. (2019), Thomas et al. (2020), and Erdem Büyükkiraz and Kesmen (2022)
Lactoferrins	LF-6	α-helix^1^	*Escherichia, Staphylococcus*	55	7	20	Jiang et al. (2016)
Penaeidins	rCHP	α-helix^1^	*Bacillus, Escherichia, Klebsiella, Micrococcus, Staphylococcus*	30	6.82	56	Li et al. (2005)
Scygonadins	CKS-Scygonadin Scygonadin (pET)	Mixed^1^	*Aeromonas, Bacillus, Corynebacterium, Escherichia, Micrococcus, Staphylococcus, Vibrio*	36	0.9	102	Peng et al. (2010)
Sericins	Sericin	AlphaFold2 Colaboratory was not able to predict a structure	*Escherichia, Staphylococcus*	53	7	96	Thomas et al. (2020)
Trp-analogs	recTritrp	α-helix^1^	*Escherichia*	27	4	15	Arias et al. (2016)
Vasoactive intestinal peptide (VIP)	Thioredoxin (Trx)-VIP8	α-helix^1^	*Escherichia, Staphylococcus*	36	7	25	Xu et al. (2017)
Proline-rich peptide	rCgPrp	α-helix^1^	*Bacillus, Listeria, Staphylococcus*	47	2.01	38	Erdem Büyükkiraz and Kesmen (2022)

^1^AMP structure predicted by AlphaFold2 Colaboratory; ^2^AMP structure deposited in the Protein Data Bank (PDB). Ratio of hydrophilic residues/total number of residues, net charge and N of residues were calculated using the online tool: https://www.bachem.com/knowledge-center/peptide-calculator/.

The 34 articles described 46 AMPs, which were divided into 18 different AMP families. Among these, the most abundant families were Defensins and Hepcidins, which had 10 and 7 different AMPs, respectively. The overall yields ranged between 0.5 mg/L and 2,700 mg/L.

Twenty-three different bacterial genera were used in the analyzed studies, and the most used were *Escherichia*, *Staphylococcus*, *Bacillus*, *Salmonella* and *Pseudomonas*. Regarding the MIC values, the distribution of data was very wide, depending on the AMP and the microorganism used for the determination, ranging between 0.125 μg/mL and > 1,152 μg/mL.

### 3.3 Risk of bias

The results of the ToxRTool are included in Supplementary Table S1. Five studies were ranked as “reliable without restrictions” as most of the required information was provided. The remaining 29 studies were lacking at least one of the red criteria”. In this situation, ToxRTool categorized these studies as “not reliable”, however, the final judgment was given by the evaluators and in some cases our judgment was discordant with the tool. Eventually, 19 studies were considered “reliable with restrictions” and 10 “not reliable”. In particular, those reliable with restrictions were including studies lacking explicit information about the use of positive and negative controls in the MIC protocol. However, since these controls are required by default in the application of standard MIC protocols (ISO 20776-1 or Disk diffusion according to EUCAST or CLSI), it was assumed that if only this information was missing, most certainly it was just omitted from the manuscript. In fact, not many studies reported the use of positive or negative controls (8 and 15, respectively). The remaining 10 studies were confirmed “not reliable” because of a combination of missing information or inconsistent parameters with the standard MIC protocols, such as inoculum size, controls, incubation time and temperature.

### 3.4 AMP families and targeted bacterial genera and relative frequency of MIC tested

**Figure 3:**
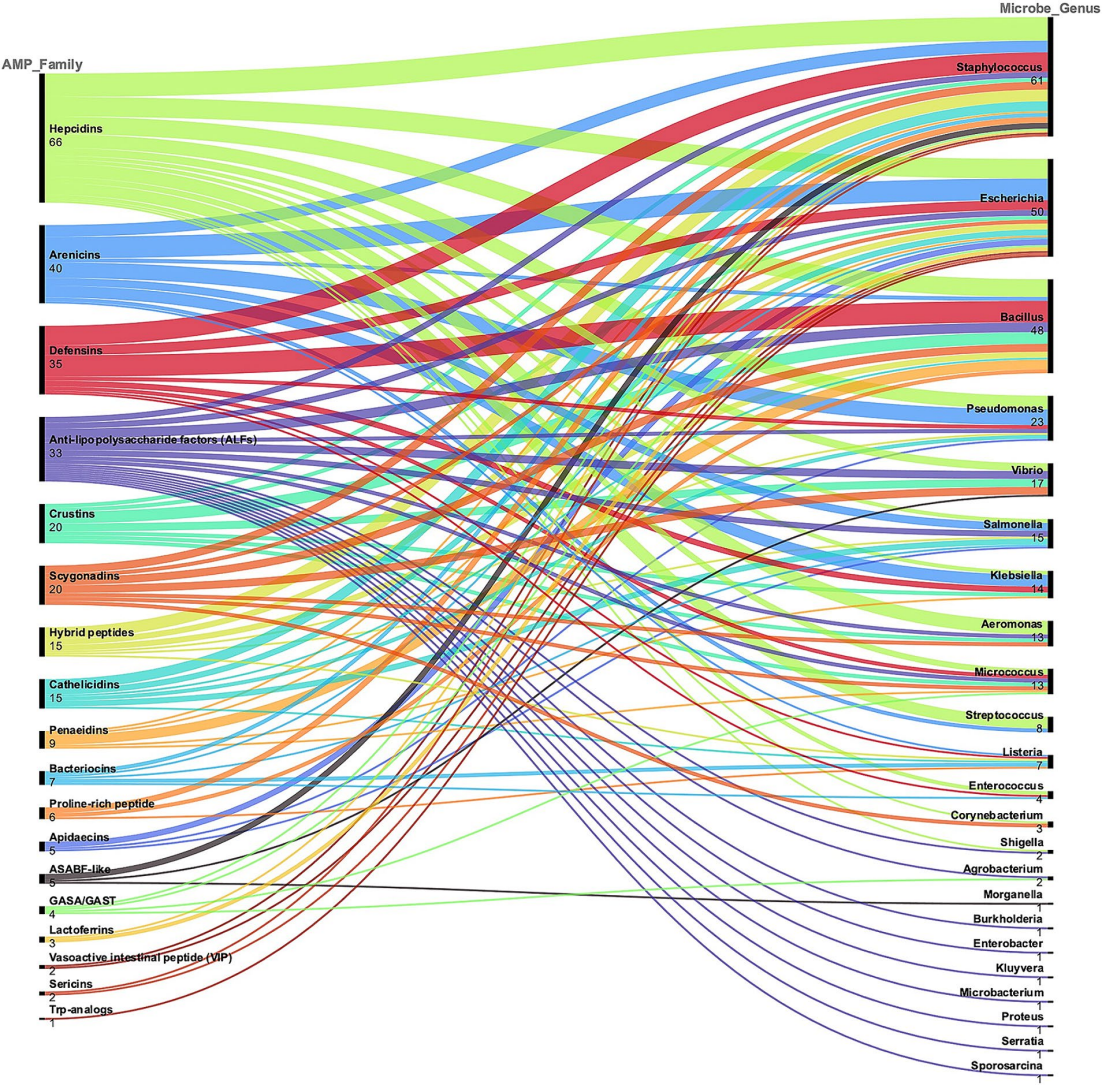
A classification of the AMP families described in the selected literature is represented, which serves as a demonstration of the diversity and complexity of these molecules. AMP families are ranked based on the total number of MIC values reported. The corresponding genus of the bacteria tested is also represented and the alluvial chart shows the connection between AMP families and microbe genus. This general categorization was used to select the most represented AMP families and targeted microorganisms.

Overall, Figure 3 shows the alluvial chart reporting the correspondence between the AMP families and the targeted bacterial genera. In this review analysis, the four most abundant AMP families were Hepcidins (66 MIC values available, in 4 studies), Arenicins (40 MICs in 3 studies), Defensins (35 MICs in 7 studies) and Anti−lipopolysaccharide factors (ALFs, 33 MICs in 3 studies). The most tested bacterial genera were *Staphylococcus*, *Escherichia*, *Bacillus* and *Pseudomonas*, with 61, 50, 48 and 23 MIC values, respectively.

### 3.5 Yield (mg/L), targeted bacterial genus and MIC range (μg/mL) of the most reported AMP families

**Figure 4:**
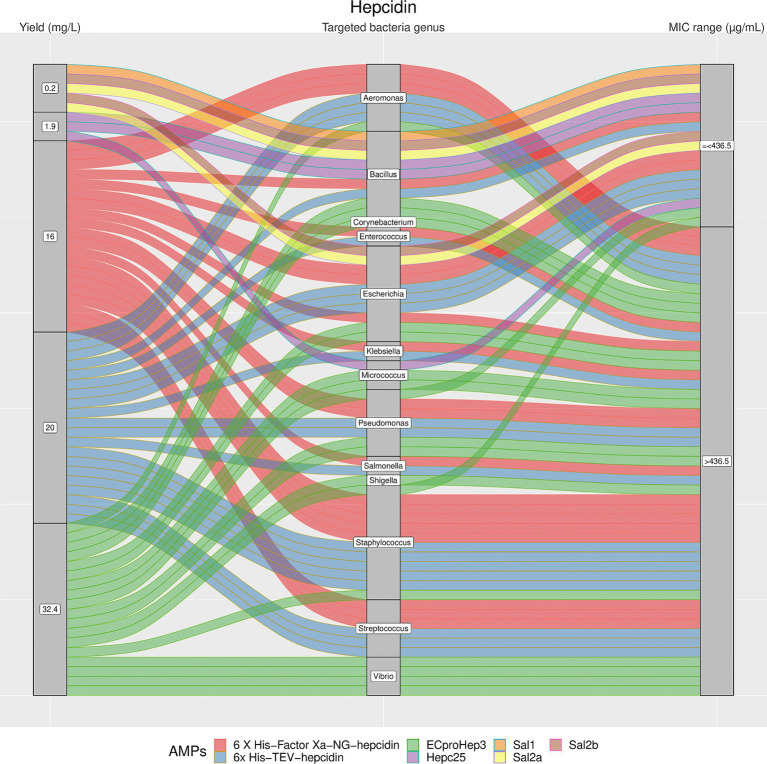
The alluvial chart visually describes the Hepcidins family by tracing their journey from production yield to their microbial targets and effectiveness. Starting from the left, the chart records the yield of each Hepcidin in mg/L, illustrating the diversity in production efficiency among the family members. This flows into the central section, which outlines the various bacterial genera targeted by these peptides, highlighting the broad spectrum of their antimicrobial activity. On the right, the minimum inhibitory concentration (MIC) range in μg/mL is reported, describing the antimicrobial activity of Hepcidins against these bacterial targets. A unique color is assigned to each AMP, thereby enabling readers to follow the story of each peptide across the chart. This figure provides a comprehensive overview of the Hepcidins’ antimicrobial capabilities and emphasizes the potential of these peptides in developing new antibacterial therapies.

**Figure 5:**
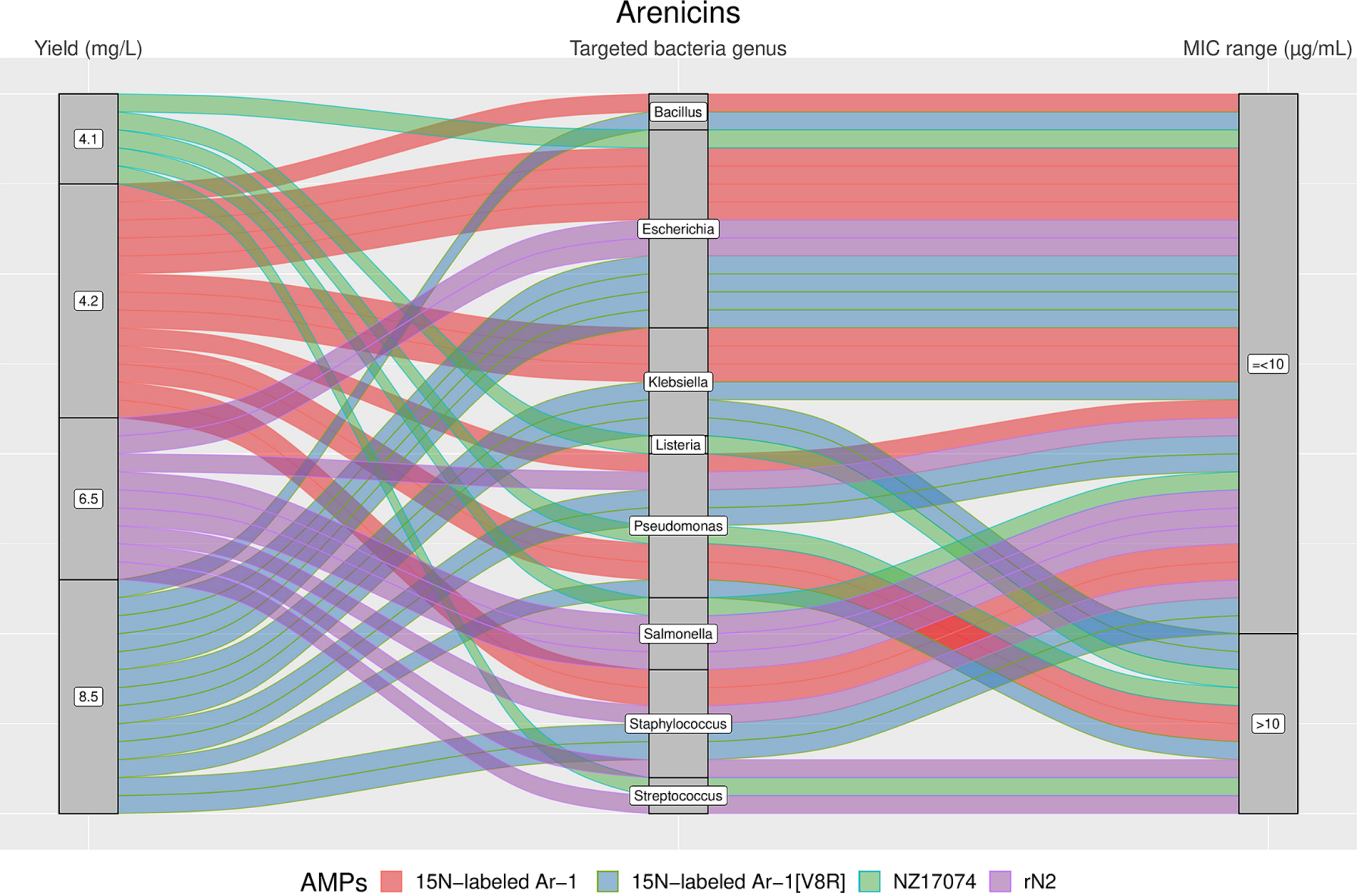
The alluvial chart visually describes the Arenicins family by tracing their journey from production yield through to their microbial targets and effectiveness. Starting from the left, the chart records the yield of each Arenicin in mg/L, illustrating the diversity in production efficiency among the family members. This flows into the central section, which outlines the various bacterial genera targeted by these peptides, highlighting the broad spectrum of their antimicrobial activity. On the right, the minimum inhibitory concentration (MIC) range in μg/mL is reported, describing the antimicrobial activity of Arenicins against these bacterial targets. A unique color is assessed to each AMP, thereby enabling readers to follow the story of each peptide across the chart. This figure provides a comprehensive overview of the Arenicins’ antimicrobial capabilities and emphasizes the potential of these peptides in developing new antibacterial therapies.

**Figure 6:**
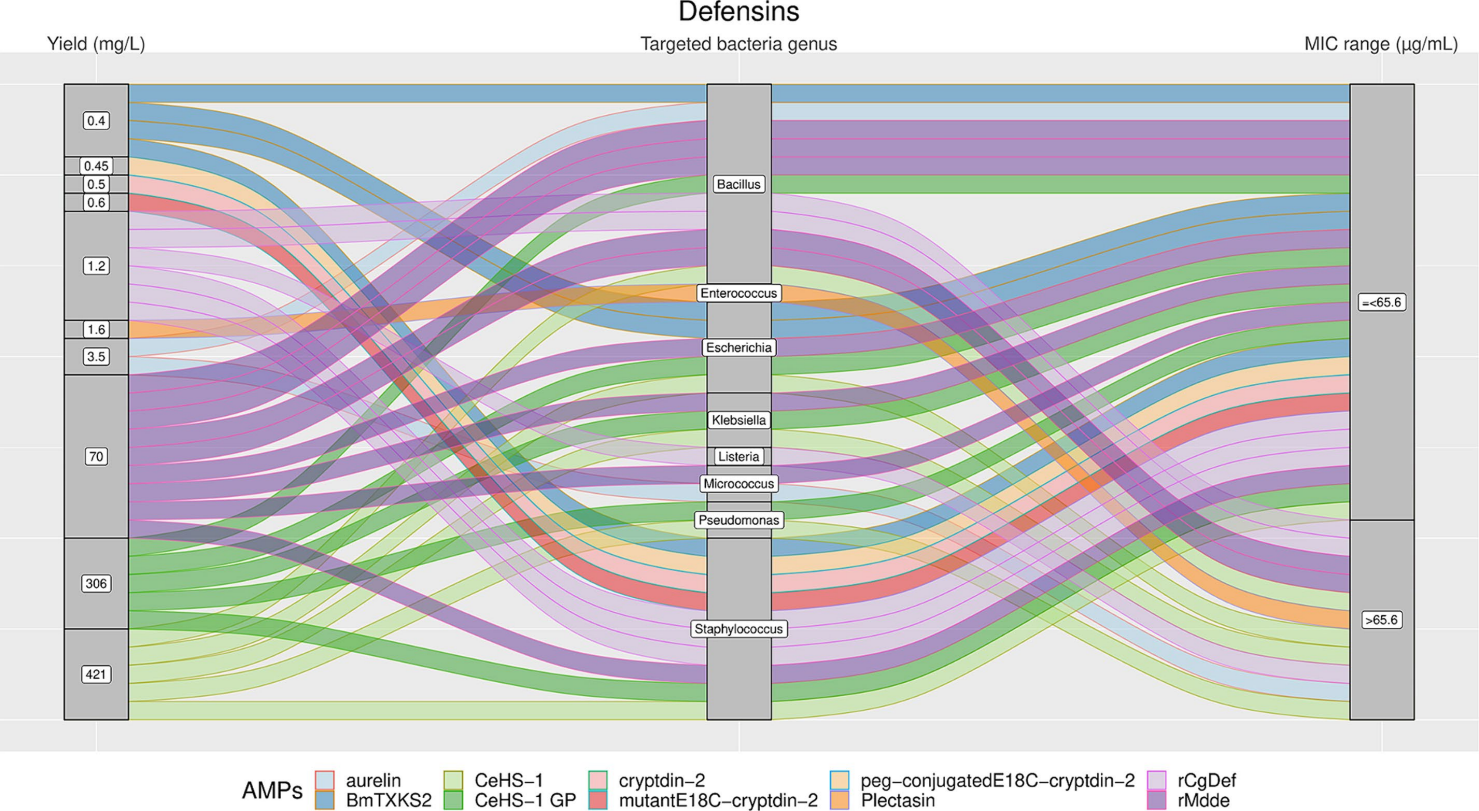
The alluvial chart visually describes the Defensins family by tracing their journey from production yield through to their microbial targets and effectiveness. Starting from the left, the chart records the yield of each Defensin in mg/L, illustrating the diversity in production efficiency among the family members. This flows into the central section, which outlines the various bacterial genera targeted by these peptides, highlighting the broad spectrum of their antimicrobial activity. On the right, the minimum inhibitory concentration (MIC) range in μg/mL is reported, describing the antimicrobial activity of Defensins against these bacterial targets. A unique color is assessed to each AMP, thereby enabling readers to follow the story of each peptide across the chart. This figure provides a comprehensive overview of the Defensins’ antimicrobial capabilities and emphasizes the potential of these peptides in developing new antibacterial therapies.

**Figure 7:**
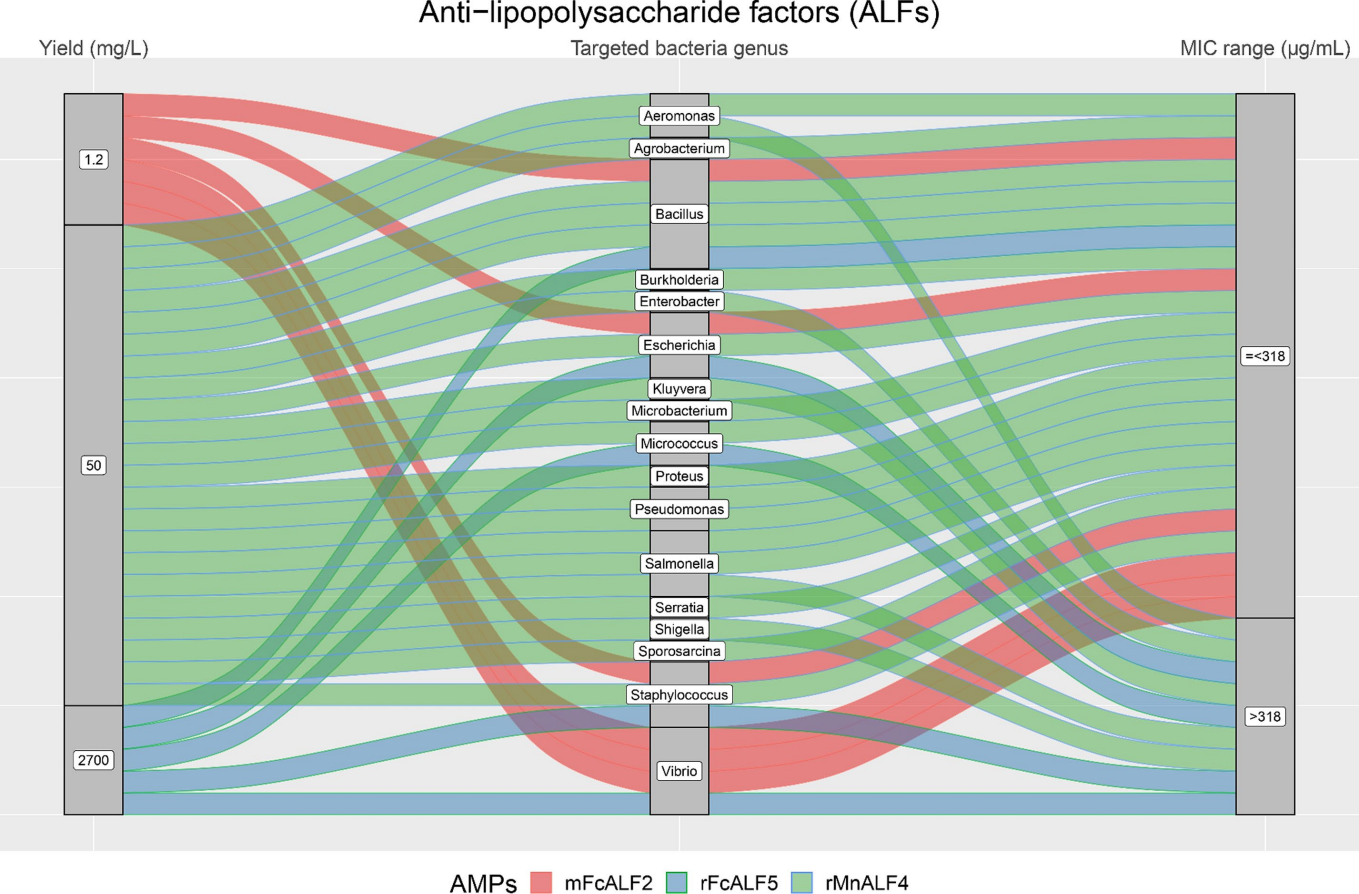
The alluvial chart visually describes the ALFs family by tracing their journey from production yield through to their microbial targets and effectiveness. Starting from the left, the chart records the yield of each ALF in mg/L, illustrating the diversity in production efficiency among the family members. This flows into the central section, which outlines the various bacterial genera targeted by these peptides, highlighting the broad spectrum of their antimicrobial activity. On the right, the minimum inhibitory concentration (MIC) range in μg/mL is reported, describing the antimicrobial activity of ALFs against these bacterial targets. A unique color is assessed to each AMP, thereby enabling readers to follow the story of each peptide across the chart. This figure provides a comprehensive overview of the ALFs’ antimicrobial capabilities and emphasizes the potential of these peptides in developing new antibacterial therapies.

From Figures 4–7, we reported the aforementioned four most analyzed AMP families with their single AMP production yield and antimicrobial activities (MIC values) plotted against different target bacteria. Between the selected AMP families, Arenicins showed the highest antimicrobial activity in terms of lowest MICs, with a 75^th^ percentile of 10 μg/mL, followed by Defensins (65.6 μg/mL), ALFs (318 μg/mL) and Hepcidins (436.5 μg/mL).

The results showed a high heterogeneity in terms of yield, as shown in Figures 4–7. Specifically, Figure 4 shows that Hepcidins were produced with yields between 0.2 (Sal2a and Sal2b) and 32.4 mg/L (ECproHep3). MIC values ranged between <18 μg/mL for *Pseudomonas stutzeri* (ECproHep3) and > 1,152 μg/mL for various specific bacteria (ECproHep3). In Figure 5, the Arenicins were produced between 4.1 and 8.5 mg/L. The lowest MIC value was 0.125 μg/mL against *Salmonella enteritidis* CVCC3377 (rN2), while the highest MIC values were > 16 μg/mL against *L. ivanovii* and *S. suis* (NZ17074). On two strains of *K. pneumoniae* and one strain of *P. aeruginosa* (15 N-labeled Ar-1[V8R]), Arecinins resulted in a MIC value of 20.25 μg/mL. The yield of Defensins (Figure 6) was between 0.4 (BmTXKS2) and 421 mg/L (CeHS-1). The MIC values for *B. subtilis* (rMdde) and *P. aeruginosa* 27,853 (CeHS-1) ranged from 4.14 μg/mL to >128 μg/mL.

As shown in Figure 7, the yield of ALFs ranged from 1.2 to 2,700 mg/L and the AMP with the highest yield (rFcALF5) also resulted in high MICs against the bacterial strains tested. The lowest MIC value of ALFs was 30 μg/mL against *B. subtilis* (rMnALF4) and the highest values were > 516 μg/mL against *E. coli* and *S. epidermidis* (rFcALF5).

**Figure 8:**
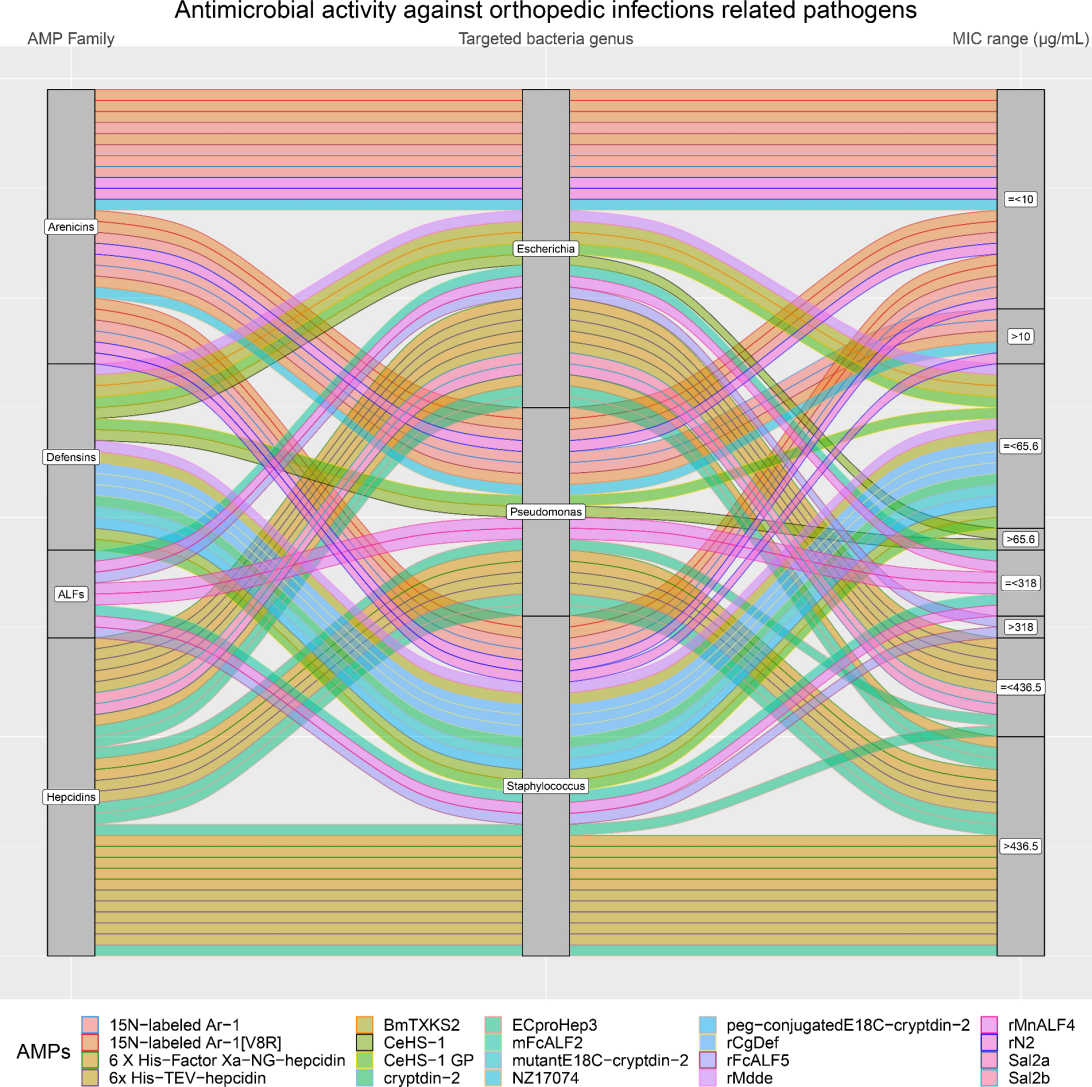
In orthopedic infections, three bacteria genera are the most represented (*Staphylococcus*, *Pseudomonas* and *Escherichia*). The alluvial chart illustrates the efficacy of various AMP families (on the left side: Arenicins, Defensins, ALFs, and Hepcidins) in inhibiting these bacteria genera. On the right side of the chart, minimum inhibitory concentration (MIC) values are categorized as either greater than (>) or less than or equal to (≤) the 75^th^ percentile, with the following thresholds: Arenicins at 10 μg/mL, Defensins at 65.6 μg/mL, ALFs at 318 μg/mL, and Hepcidins at 436.5 μg/mL. The legend at the bottom shows each AMP considered in the chart.

Moreover, the antimicrobial activity of the four selected AMP families against the most common pathogens involved in orthopedic infections (*E. coli, Pseudomonas*, and *Staphylococcus*) was visually analyzed. Figure 8 confirms that Arenicins have the highest antimicrobial activity and the lowest MICs compared to the other three AMP families. In particular, almost all of the AMPs of the Arenicins family included in this study showed MIC values below 10 μg/mL against *E. coli*, *Pseudomonas*, and Staphylococcal strains. The only exceptions were 15 N-labeled Ar-1 against *Pseudomonas_aeruginosa*_XDR_CI_1049_ESBL+_MBL+, and *Pseudomonas_aeruginosa*_XDR_CI_236_ESBL+_MBL+ (MIC of 20.12 μg/mL), 15 N-labeled Ar-1 [V8R] against *Pseudomonas_aeruginosa*_XDR_CI_236_ESBL+_MBL+ (MIC of 20.25 μg/mL) and rN2 against MRSA_ATCC_43300 (MIC of 32 μg/mL).

On the other hand, Arenicins were produced on average at lower levels than the other three AMP families (4.1 to 8.5 mg/L), although there were no statistically significant differences between the four AMP families’ yields (*p* > 0.05). Defensins family included three AMPs produced in high yields (rMdde, 70 mg/L; CeHS-1 GP, 306 mg/L and CeHS-1, 421 mg/L), which showed an antimicrobial activity with MIC values higher than the ones produced by the Arenicins, but lower than Hepcidins and ALFs. In particular, rMdde showed a MIC of 23 μg/mL against *E. coli* and *S. aureus*, CeHS-1 GP displayed a MIC of 64 μg/mL against *E. coli* ATCC 25922, *P. aeruginosa* 27853 and *S. aureus* ATCC 25293 and CeHS-1 exhibited a MIC of 64 μg/mL against *S. aureus* ATCC 25293 and a MIC of 128 μg/mL against *E. coli* ATCC 25922.

## 4 Discussion

Orthopedic infections are highlighted as a major concern, emphasizing the need for effective treatment. The antibiotic resistance as a “silent pandemic” draws attention to the gravity of the issue in the context of orthopedic infections (Mendelson et al., 2022). This paves the way for the main focus of the study, which is the role of AMPs in addressing these challenges.

The findings of this comprehensive review shed light on the antimicrobial potential and production yield of recombinant AMPs, offering valuable insights into their applicability as potential therapeutic agents against common bacterial pathogens involved in clinical infections. To date, this is the first systematic literature review focusing specifically on recombinantly produced AMPs. The primary goal of this study was to identify which AMP families exhibited the highest antimicrobial efficacy and production yields after purification, and the results provide a nuanced understanding of the relationships between these factors.

The PEO model allowed for the systematic extraction of data from a wide array of studies, ensuring a comprehensive assessment of recombinant AMPs performance. The stringent exclusion criteria, which eliminated studies reporting only one of the two essential data sets (yield and MIC), those utilizing non-purified AMPs, and studies deviating from the CLSI guidelines for MIC assessment, enhanced the overall reliability of the analyzed data.

The 34 selected studies collectively described 46 distinct recombinant AMPs, which were classified into 18 unique AMP families, and targeting 23 bacterial genera. Among AMPs, Defensins and Hepcidins stood out as the most abundant families, collectively contributing 18 different AMPs.

The use of the ToxRTool to assess the risk of bias in the included studies provided an additional layer of scrutiny. While only five studies achieved a “reliable without restrictions” classification, the majority of the studies were categorized as “reliable with restrictions” due to gaps in reporting, particularly concerning positive and negative controls in MIC protocols. It would be a good practice, however, to always mention the use of positive and negative controls in all experiments, even when referring to a previous publication in the Methods section. Other issues raised in the risk of bias analysis regarding heterogeneity in the inoculum size, incubation temperature and time. It is well known that changes in the inoculum size determine variations in the MICs (Loffredo et al., 2021), and it would be good practice to always follow the CLSI guidelines regarding time and temperature of incubation, if the bacterial species used in the experiment are listed in the guidelines, to keep the protocol as much standardized as possible, for the sake of providing data comparable with the rest of literature. Unfortunately, a poor reporting prevents the interpretation of many studies even if they are well conducted. We call for a better planning of protocols, because all necessary information for implementation needs to be reported.

The analysis of AMP families’ antimicrobial activity and production yields against various target bacteria elucidated several key insights. The alluvial charts demonstrated the relationship between AMP families and bacterial targets is an effective way to visualize complex data and highlighted the most frequently tested AMP families. Hepcidins, Arenicins, Defensins, and Anti-lipopolysaccharide factors (ALFs) result as the most prominent candidates. This information is valuable for researchers and clinicians seeking to prioritize AMP families for further investigation or therapeutic development.

The observed heterogeneity in yield (as illustrated in Figures 4–7) emphasizes the need for optimization in the expression and purification processes. The variability in peptides production across different studies and AMP families, spanning from 0.5 mg/L to 2,700 mg/L, highlighted that, despite the multiple approaches used for AMPs production, no standardized methods exist today to reliably provide the high required yield. Further optimization of production methods to ensure cost-effectiveness for clinical applications and commercialization, is required. While certain AMPs within a family may exhibit promising antimicrobial activity, achieving consistent high yields remains a challenge. Cell free synthesis could represent a promising strategy in AMPs production, not requiring plasmids and thus avoiding limitations in molecular design; moreover, recent advancements in the protein purification methods from these cell free system platforms, could provide scalable protein synthesis (Gregorio et al., 2019; Khambhati et al., 2019). Noteworthy, an improvement in activity and stability of AMPs, could allow lower AMPs concentrations and dosage during treatment: the conjugation of AMPs with other peptides or proteins could improve the resistance to pH and temperature, and the antimicrobial activity (Toyohara et al., 2019), thus making cheaper the AMPs utilization. Protein engineering studies based on computational methods are being employed for the rational design of new AMPs with improved activity and easier production. Moreover, advances in machine learning methods allowed to screen and design candidate AMPs with different 3D-structure and function for experimental evaluation (Hiss et al., 2010; Boone et al., 2021). In a similar way, genetic algorithms and predictive methods can be used to predict the antimicrobial activity (Kliger, 2010; Fjell et al., 2011). *In silico* tools allow to assign to each peptide an antimicrobial score linearly correlated to the antimicrobial potency of the AMP on the basis of their amino acid composition. In detail, the value is correlated to the product CmHnL, where C is the net charge of the AMP, H is a measure of its hydrophobicity, L its length, and the exponents “m” and “n” are two strain dependent variables which determine the relative contribution of charge and hydrophobicity (Pane et al., 2017). In fact, even if a net negative charge is present on both sides of the bacterial membrane, each strain shows peculiar lipid composition: for example negatively charged phospholipids such as cardiolipin and phosphatidylglycerol can vary from 20% in *E. coli* to almost 100% in Staphylococcal strains (Epand and Epand, 2011). Accordingly, it is unlikely that a single AMP could interact in a similar way with such different bilayers. As a result, the combination effects of AMPs could improve the efficacy of the treatment, thus allowing to save costs of production and, at the same time, reduce the side effects. Different studies on AMPs interaction suggest synergism as a common phenomenon mainly due to the heterogeneity in the targeted bacteria genus, as illustrated in Figures 4–7 (Yu et al., 2016). The emphasis on MIC values and the categorization of AMP families based on antimicrobial activity provide insights to identify the most effective AMPs against specific bacteria. Similarly to the yield, the wide range of reported MIC values (from 0.125 μg/mL to >1,152 μg/mL) reflects the diversity in AMPs efficacy. In general, despite lower yields, the high efficacy of Arenicins, as indicated by their lower MIC values, is an important finding, suggesting their potential as effective antimicrobial agents. On the other hand, Defensins were produced with higher yields, and their MIC values were slightly higher than the Arenicins, making them still good antimicrobial candidates.

The specific focus on pathogens commonly involved in orthopedic infections is highly relevant, given the increasing concern about antibiotic-resistant bacteria in such settings. The most prevalent efficacy of Arenicins against *E. coli*, *Pseudomonas*, and *Staphylococcus aureus* is particularly noteworthy, aligning with the need for more effective treatments in clinical settings. Defensins also were particularly relevant in this study. In their case, higher yields were observed, with MIC values, against orthopedic infections relevant pathogens, slightly higher than the Arenicins. Based on the data collected and explored in this review, both Arenicins and Defensins would require more attention in future studies. *Staphylococcus aureus*, *E. coli* and *Pseudomonas*, the most common pathogens found in orthopedic infections, were inhibited by low concentrations of both AMP families, however, no data were found in the selected papers about *S. epidermidis*, another important Staphylococcal species involved in orthopedic infections. The biofilm formed by *S. epidermidis* is a subtle threat because it can contribute to the development of antimicrobial resistance in the infected areas, affecting the quality of life in patients, with lower possibilities of a positive outcome (Hischebeth et al., 2019; Bottagisio et al., 2023). It is important, for such reasons, to assess the efficacy of these AMPs also against additional microorganisms that are responsible of orthopedic infections. Furthermore, the production of Arenicins needs optimization, to increase their yields without compromising their antimicrobial potential. For both AMP families, more data are required to confirm their antimicrobial potential, also against different clinical isolates. Eventually, their stability, cytotoxicity and hemolytic activity need to be assessed, with standardized protocols, to create a larger dataset that proves their usability in the further steps, such as *in vivo* models.

In summary, this systematic review provides a comprehensive assessment of recombinant AMPs antimicrobial potential and production yields, offering a valuable resource for researchers and clinicians engaged in coping antibiotic-resistant infections. While challenges and variations exist, the study highlights the promise of AMPs as a potential avenue for addressing the urgent issue of antimicrobial resistance in clinical settings. Further research and development in this field hold the potential to unlock novel treatments for a wide range of infections, including those related to orthopedic procedures.

## Data availability statement

Publicly available datasets were analyzed in this study. This data can be found here: https://osf.io/2uq4c/.

## Author contributions

VP: Conceptualization, Data curation, Formal analysis, Investigation, Methodology, Supervision, Writing – original draft, Writing – review & editing, Software. ER: Conceptualization, Data curation, Formal analysis, Writing – original draft, Writing – review & editing. EM: Data curation, Writing – review & editing. SG: Formal analysis, Methodology, Writing – review & editing. GC: Formal analysis, Methodology, Writing – review & editing. SB: Formal analysis, Methodology, Writing – review & editing. AL: Conceptualization, Data curation, Formal analysis, Writing – original draft, Writing – review & editing, Funding acquisition, Methodology, Project administration, Supervision.
